# Moderate‐intensity aerobic exercise attenuates obesity‐induced changes in perivascular adipose tissue in female mice

**DOI:** 10.14814/phy2.70506

**Published:** 2025-08-22

**Authors:** Teresa M. Da‐Re, Guilherme A. dos Santos, Luiza H. Rossi, Camila B. Risso, Sílvio R. Consonni, Maria A. Delbin

**Affiliations:** ^1^ Departamento de Biologia Estrutural e Funcional, Instituto de Biologia Universidade Estadual de Campinas (UNICAMP) Campinas São Paulo Brazil; ^2^ Departamento de Bioquímica e Biologia Tecidual, Instituto de Biologia Universidade Estadual de Campinas (UNICAMP) Campinas São Paulo Brazil

**Keywords:** exercise, female, inflammation, obesity, perivascular adipose tissue, senescence, vascular function

## Abstract

The goal of the study was to investigate the effects of aerobic exercise training on inflammatory, oxidative stress, and cellular senescence biomarkers, along with the evaluation of morphology and anti‐contractile response of perivascular adipose tissue of the thoracic aorta (tPVAT) in female mice (C57BL6/JUnib) divided into sedentary (SD), trained (TR), obese sedentary (OB‐SD), and obese trained (OB‐TR). In the OB‐SD group, serum glucose, leptin, TNF‐α, and malondialdehyde were increased, linked with enlarged lipid droplets, decreased adiponectin content in tPVAT, and enhanced vascular β‐galactosidase activity accompanied by alterations in the anti‐contractile response of tPVAT to serotonin (5‐HT) compared with the SD group. Aerobic exercise training was effective in reducing serum leptin, TNF‐α, and malondialdehyde in the OB‐TR group, with smaller lipid droplets and increased adiponectin in the tPVAT, and attenuation of vascular β‐galactosidase activity with beneficial effects in the vascular response to 5‐HT. This study highlights a significant phenotypic change of the tPVAT in obese female mice with increased vascular senescence and dysregulation of the anti‐contractile response, systemic inflammation, and oxidative stress. Aerobic exercise training effectively mitigated some of the obesity‐induced changes in the tPVAT, partially restoring the anti‐contractile response to 5‐HT and reducing circulatory leptin, TNF‐α, and malondialdehyde.

## Graphical abstract

Aerobic exercise attenuates obesity‐induced tPVAT dysfunction by reducing inflammation, oxidative stress, and restoring anti‐contractile vascular response in female mice.

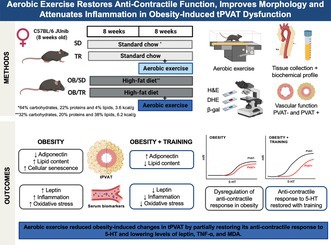

## 1 INTRODUCTION

Until the early 1990s, women were largely excluded from clinical trials, and sex‐specific analyses were not extensively studied until more recently. As a result, the prevention, diagnosis, and treatment of chronic diseases, such as cardiovascular disease (CVD) in women, have primarily relied on findings from studies conducted on male animal models or men (Regensteiner & Reusch, 2022), creating potential barriers and unintended consequences. Approximately 878 million adults worldwide are currently living with obesity, and by 2035, this number is expected to approach 2 billion. The prevalence is higher in women, with 18.5% living with obesity, compared to 14% of men (World Obesity Federation, 2024). Obesity carries a unique disease burden for women and is influenced by a range of biological, environmental, and cultural factors (Azarbad & Gonder‐Frederick, 2010).

Perivascular adipose tissue (PVAT) is a special type of ectopic fat deposit that surrounds most blood vessels and exerts both endocrine and paracrine functions due to its ability to secrete adipokines and anti‐inflammatory mediators (Man et al., 2022; Sowka & Dobrzyn, 2021). The PVAT exhibits regional phenotypic heterogeneity; interestingly, it can be categorized as white adipose tissue (WAT), brown adipose tissue (BAT), or a mixture of both (beige adipose tissue), with varying proportions depending on the anatomical location, resulting in differences in the regulation of specific adipokine signaling pathways in regional PVAT (Fitzgibbons et al., 2011; Guo et al., 2024; Padilla et al., 2013).

Under physiological conditions, PVAT plays an important role in the regulation of vascular function, acting on both vascular smooth muscle cells (VSMCs) and endothelial cells (ECs) through endothelium‐dependent and endothelium‐independent mechanisms (Maenhaut & Van de Voorde, 2011; Man et al., 2022). Several studies conducted in male rodents have demonstrated the anti‐contractile effect of PVAT in response to endothelin‐1 (ET‐1) in rat iliac and mesenteric arteries (Cruz‐López et al., 2024; Szasz & Webb, 2012), to phenylephrine (PHE) in mouse thoracic aorta (Lazaro et al., 2024) and to serotonin (5‐HT) (Araujo et al., 2018) and angiotensin II (ANG II) (Gálvez‐Prieto et al., 2012) in rat thoracic aorta. The factors responsible for this effect are classified as adipocyte‐derived relaxing factors (ADRF) and perivascular‐derived relaxing factors (PDRF) (Dubrovska et al., 2004; Gollasch, 2012). Different responses towards the anti‐contractile effect can be observed according to the vascular bed. As presented in male rats, thoracic aorta PVAT (tPVAT) exerts an anti‐contractile effect in response to PHE, whereas this modulation is absent in the abdominal aorta PVAT (Victorio et al., 2016). This difference was associated with lower endothelial nitric oxide synthase (eNOS) expression and NO production in the abdominal region (Victorio et al., 2016). Conversely, PVAT can also induce vasoconstriction through the release of ANG II (Lu et al., 2010), superoxide anion (O_2_^−^) (Gao et al., 2006), 5‐HT, and norepinephrine (NE) (Ayala‐Lopez et al., 2014).

The energy imbalance observed in obesity results in the remodeling of adipose tissue, with hyperplasia and hypertrophy of adipocytes (Costa et al., 2018; Spalding et al., 2008). In addition to changes in the proportion of cells with age, nutritional status, and environmental conditions (Miao & Li, 2012), regional differences in PVAT are associated with distinct functions and pathophysiological outcomes (Contreras et al., 2016). Changes in its secretory profile, induced by obesity, result in pro‐inflammatory and pro‐oxidative states (de Mello et al., 2018; Ketonen et al., 2010; Marchesi et al., 2009). In obese male mice, the regional heterogeneity of PVAT becomes more pronounced, mainly due to its phenotypic transformation (Chatterjee et al., 2009; Fitzgibbons et al., 2011; Ketonen et al., 2010). Adipocytes from male mice presented an accumulation of macrophages with genes associated with increased production of pro‐inflammatory cytokines (Lumeng et al., 2007). In addition, in obese male mice or rats, the property of PVAT to regulate the vascular contraction in response to 5‐HT and/or PHE is altered, including loss (da Costa et al., 2017) or enhanced anti‐contractile response (Araujo et al., 2018).

Inflammation and oxidative stress are important mediators in the association between obesity and aging. Studies have shown that obese individuals exhibit increased senescent cells in adipose tissue (Conley et al., 2020; Tchkonia et al., 2010). The chronic inflammatory environment increases the production of reactive oxygen species (ROS), which can overload the antioxidant defenses, resulting in oxidative stress (Ou et al., 2022). Thus, the pro‐inflammatory and pro‐oxidative profile established by obesity stimulates inflammation and may impair stem cell renewal capacity, thereby consolidating a senescence‐associated secretory phenotype (SASP) that is independent of chronological age (Palmer et al., 2022). The remodeling of PVAT in obesity is associated with increased production and release of contractile factors and a reduction in relaxant factors derived from PVAT (Ahima, 2009; Ou et al., 2022; Zhou et al., 2021).

Vascular senescence is characterized by vascular dysfunction resulting from the cellular senescence of vascular wall cells (ECs and VSMCs) (Wang et al., 2009). A study demonstrated that diet‐induced obesity in male mice causes aortic ECs senescence and dysfunction through long‐term intermittent activation of Akt (Wang et al., 2009). Recent literature supports that ECs senescence is regulated by multiple factors, including ET‐1, nitric oxide synthase (NOS), and oxidative stress (Bloom et al., 2022). Specifically, eNOS activity promotes endothelial senescence (Han & Kim, 2023). The underlying mechanisms are not fully elucidated, and other factors may also contribute to obesity‐induced vascular senescence, representing an additional risk factor for the development of CVD (Li et al., 2025).

Aerobic exercise training is among the strategies and therapies that can influence the cardiometabolic complications observed in obesity (Lavie et al., 2015). Moderate‐intensity aerobic exercise, when performed continuously, promotes beneficial effects both in the prevention and treatment of cardiovascular and endocrine‐metabolic diseases (Ciolac & Guimarães, 2004; Ko et al., 2023; Shi et al., 2022). It has been demonstrated that acute moderate‐intensity exercise stimulates important anti‐inflammatory properties, reducing inflammation in the stromal vascular fraction of epididymal adipose tissue in male rats (Oliveira et al., 2013). Regular moderate‐intensity aerobic exercise reduced body fat, adipocyte hypertrophy, and consequently, the number of inflammatory cells in the epididymal adipose tissue of male mice (Kawanishi et al., 2013). Another key process linked to the effects of physical exercise is the browning of adipose tissue. An increased presence of brown or beige adipocytes is considered a marker of a metabolically healthy phenotype (Min et al., 2016). A study in male rats indicated that moderate‐intensity aerobic training enhanced sympathetic adrenergic tone, restored the distribution of lipid droplets, and boosted uncoupling protein 1 (UCP‐1) expression in brown adipocytes within the visceral adipose tissue (De Matteis et al., 2013).

Most studies have been conducted in male models; consequently, sex‐specific clinical guidelines are largely absent. Optimal care for females should incorporate an understanding of sex specificities into diagnosis and therapeutic guidelines. Thus, this study aimed to investigate the effects of moderate‐intensity aerobic exercise training on phenotypic, inflammatory, oxidative stress, cellular senescence, and anti‐contractile response changes in tPVAT in obese female mice.

## 2 MATERIALS AND METHODS

### 2.1 Experimental animals

**FIGURE 1:**
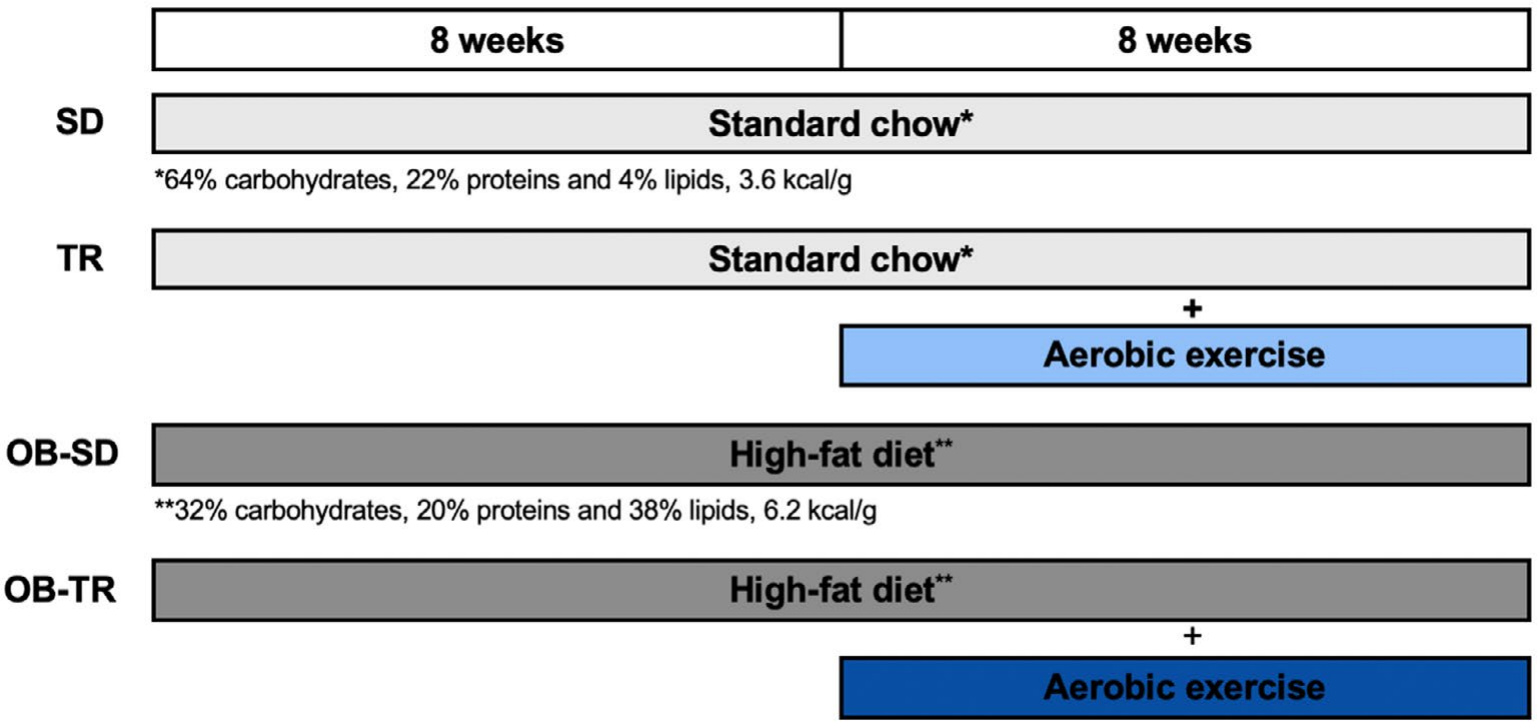
Experimental protocol. Eight‐week‐old female mice were divided into the following groups: sedentary (SD), trained (TR), obese sedentary (OB‐SD), and obese trained (OB‐TR). After 8 weeks on a high‐fat diet, the TR and OB‐TR groups initiated the aerobic exercise program consisting of 60‐min sessions, 5 days a week, for 8 weeks. The respective diets (standard chow or high‐fat diet) were maintained throughout the entire 16‐week protocol.

The study was approved by the Ethics Committee for Animal Use (CEUA 5849‐1) at the Universidade Estadual de Campinas (UNICAMP), as stated by the Brazilian Society of Laboratory Animal Science. Female C57BL/6JUnib mice (4–5 weeks, weighing 17–21 g) from the Animal Care Facility of UNICAMP were maintained in a room at 20–21°C with a normal light/dark cycle. Food and water were provided ad libitum to all animals. The animals were housed in three or four animals per cage and, at 8 weeks of age, were separated into experimental groups: sedentary (SD), trained (TR), obese sedentary (OB‐SD), and obese trained (OB‐TR). For 16 weeks, the SD and TR groups were fed standard chow (64% carbohydrates, 22% proteins, and 4% lipids, providing 3.6 kcal/g, Nuvilab CR1, Brazil). In contrast, the OB‐SD and OB‐TR groups were fed a high‐fat diet (32% carbohydrates, 20.3% proteins, and 38% lipids, providing 6.2 kcal/g, PragSoluções Biociências, Brazil). The animals in the TR and OB‐TR groups initiated aerobic exercise training after 8 weeks of study, which was maintained until the end of the protocol for an additional 8 weeks (Figure 1). Body weight, food, and water intake were measured weekly throughout the study.

### 2.2 Aerobic exercise protocol

The animals were trained between 6:00 and 8:00 a.m. on a treadmill designed for small animals (AVS Projetos, Brazil). In the week before the start of the aerobic exercise program, the animals from the TR and OB‐TR groups underwent an adaptation period on the treadmill, during which the imposed speed ranged from 3 m per minute (m/min) to 9 m/min. The duration increased from 20 min on the first day to 30 min on the last adaptation day. The exercise program consisted of sessions of 60 min/day, 5 days/week, at a 0% grade and at 50%–60% of the maximal speed, determined using an acute incremental test previously described (Ferreira et al., 2007), for 8 weeks. In the first week of the training program for the TR group, the duration and speed started at 9 m/min for 30 min and were progressively increased to 12 m/min for 60 min. The OB‐TR group started at 6 m/min for 30 min and, by the end of the week, increased to 9 m/min for 60 min. From the second to eighth week, the training program consisted of 10 min at 40% of maximal speed, 40 min at 50%–60% of maximal speed, and 10 min at 40% of maximal speed (total of 60 min/day). In the TR group, the speed corresponding to 50%–60% of the maximal speed range was 14.3 m/min to 17.2 m/min, and for the OB‐TR group was 11.7 m/min to 14.1 m/min. All groups of animals were submitted to acute incremental exercise testing on the treadmill during the last week of the study to evaluate the effectiveness of the training program.

### 2.3 Intraperitoneal glucose and insulin tolerance tests

A specific set of animals was carried out to the intraperitoneal glucose tolerance test (ipGTT) and insulin tolerance test (ipITT). Forty‐eight hours after the exercise training session, mice were fasted for 6 h (8:00 a.m. to 2:00 p.m.) for ipGTT. Baseline blood glucose was measured from the tail vein using a digital glucometer (Accu‐Check Performa Nano, Roche Diagnostics, Switzerland) and standard test strips (#06454011, Accu‐Chek Advantage, Roche Diagnostics, Switzerland) with fully conscious mice, followed by an intraperitoneal injection of glucose (2.0 g/kg body weight). Subsequent blood samples were collected at 15, 30, 60, 90, and 120 min after the injection (Andrikopoulos et al., 2008). The day after the ipGTT, the animals from the TR and OB‐TR groups performed a regular session of aerobic exercise.

In the same set of animals, 48 h after the exercise training session, mice were fasted for 2 h (8:00 a.m.–10:00 a.m.) for ipITT. Baseline blood glucose was measured from the tail vein with fully conscious mice, followed by an intraperitoneal injection of insulin (1 U/kg body weight; Humulin R, Eli Lilly, USA). Blood samples were collected at 5, 10, 15, 20, 30, and 60 min after injection (Marmentini et al., 2021; Valgas et al., 2021).

### 2.4 Blood pressure measurement

An additional set of animals was used in the experiments described below. Systolic blood pressure (SBP) and heart rate (HR) were assessed using tail‐cuff plethysmography (LE5001 Pressure meter, Panlab, Harvard Apparatus, Spain) the week before tissue collection. Before the experiment, the animals underwent a 4‐day habituation period, which involved encouraging them to enter and remain inside the restrainer, heated by a thermostat to a temperature of 33–35°C, for 5 min. On the fifth day, 6 h after the last exercise session, the animals were kept in the measurement laboratory for 1 h. Animals were restrained for 2 min before the first SBP measurement, which was considered successful when the mouse did not move and a clear pulse was observed. Twenty sequential measurements from each animal were registered. The first five measurements were not considered, and SBP was expressed as the average of the 15 measurements (Wilde et al., 2017).

### 2.5 Tissue collection and serum biochemical profile

Forty‐eight hours after the last exercise training session and the following 12 h of overnight fasting, blood glucose was measured from the tail vein using a digital glucometer (Accu‐Check Performa Nano, Roche Diagnostics, Switzerland) and standard test strips (#06454011, Accu‐Chek Advantage, Roche Diagnostics, Switzerland). Thereafter, the animals were anesthetized (ketamine, 240 mg/kg; xylazine, 30 mg/kg), and blood samples were collected by cardiac puncture and then centrifuged (1000 rpm for 15 min). Immediately after blood sample collection, animals were euthanized with an overdose of anesthetic agent and exsanguination. Thoracic aorta with PVAT, perigonadal white adipose tissue (pWAT), interscapular brown adipose tissue (iBAT), and tibia samples were collected and properly stored until the analysis was performed.

Serum levels of 17β‐estradiol (#KB30‐H1, Arbor Assays, USA), leptin (#ELM‐Leptin, RayBiotech, USA), adiponectin (#ELM‐Adiponectin, RayBiotech, USA), TNF‐α (#MHSTA50, R&D Systems, USA), and malondialdehyde (#10009055, Cayman, USA) were determined using commercial kits.

### 2.6 Histology

Thoracic aorta with PVAT, pWAT, and iBAT samples were fixed by immersion in 4% paraformaldehyde in PBS (0.1 M) at pH 7.4 and 4°C. Subsequently, the samples were washed in buffer solution, dehydrated through a series of alcohols, and embedded in Historesin (#7022‐18 500, Leica Biosystems, Germany). Sections of 3 μm were stained using the hematoxylin and eosin–floxin B 1% method (Bennett et al., 1976; Consonni et al., 2012). The materials were documented using a Nikon Eclipse 800 photomicroscope (Nikon, Japan), with a P6FL PRO digital camera (Optika, Italy), using 10× and 40× objectives. The thoracic aorta with PVAT images were quantified using the ImageJ software (National Institutes of Health, USA). The quantification of the adipose percentage in the tissue area was calculated and adapted from a previously described method (Parlee et al., 2014; Sternberg, 1983).

### 2.7 Vascular function

Thoracic aorta was placed in ice‐cold Krebs solution and cut into rings of 3 mm in the absence (PVAT−) or in the presence (PVAT+) of PVAT. The rings were mounted in a myograph chamber (model 610 M; Danish Myo Technology, Denmark) under a resting tension of 5 millinewtons (mN), as previously described (Sousa et al., 2019). The vascular maximal contraction was determined by replacing Krebs with KCl 80 mM. After that, the rings were washed with Krebs solution and the contractile‐response curves to thromboxane A_2_ analogue (U46619, 100 pm–3 μM) (#1932, Tocris, United Kingdom) and serotonin (5‐HT, 1 nM–100 μM) (#H9523, Sigma‐Aldrich, USA) were obtained. Contractile responses were calculated according to the force and length from each ring as millinewton per millimeter (mN/mm). The concentration‐response data were evaluated for a fit to a logistics function (Instat Software, GraphPad Prism, USA). The responses for each agonist are shown as the mean ± standard deviation (SD) of maximum response (E_MAX_) and potency (pEC_50_).

### 2.8 Tissue adiponectin

The PVAT was isolated from the thoracic aorta and homogenized in the Tissue Lyser LT (#85600, Qiagen, USA) in RIPA lysis buffer (#20‐188, Millipore, USA). The homogenate was centrifuged, and the supernatant was collected for protein concentration determination using Pierce BCA Protein Assay kit (#23227, Thermo Fischer, USA). Adiponectin quantification was performed using a commercial kit (#ELM‐Adiponectin, RayBiotech, USA) to which 2 μg of protein from tPVAT in a final volume of 100 μL assay diluent, previously calculated, was applied.

### 2.9 Reactive oxygen species

Thoracic aorta with PVAT was collected in a specific medium, Tissue‐Tek O.C.T. Compound (#4583, Sakura, USA); transverse sections of 10 μm were obtained, and slides were incubated in PB (0.1 M) containing diethylenetriaminepentaacetic acid (DTPA 100 μM) (#32325, Sigma Aldrich, USA) at pH 7.4 and 37°C for 10 min. Fresh buffer containing hydroethidine (DHE 2 μM) (#D1168, Thermo Fisher Scientific, USA) was topically applied, and the slides were incubated protected from light at 37°C for 30 min. Images were obtained using a microscope (Nikon Eclipse, Japan) equipped with a rhodamine filter, using a 10× objective. The fluorescence of ethidium bromide‐positive nuclei was analyzed using the ImageJ software (National Institutes of Health, USA). The results from all groups were calculated relative to the SD group (SD = 1).

### 2.10 β‐Galactosidase activity

Thoracic aorta with PVAT was collected in specific medium Tissue‐Tek O.C.T. Compound (#4583, Sakura, USA), and the quantification of senescent β‐galactosidase staining was performed using the adapted protocol of commercial kit (#9860, Cell Signaling, USA). Transverse sections of 10 μm were obtained, and slides were washed with PBS and fixed for 15 min at room temperature with the fixative solution. Subsequently, the slides were rewashed with PBS, and 120 μL of the staining solution (β‐galactosidase staining) was added to each section. The slides were incubated for 12 h in a dry incubator at 37°C (Lemos et al., 2025). Images were obtained using a Nikon Eclipse 800 photomicroscope (Nikon, Japan) with a P6FL PRO digital camera (Optika), using a 40× objective. The images were analyzed using the ImageJ software (National Institutes of Health, USA) and quantified based on the intensity of the blue staining, characteristic of β‐galactosidase. The results from all groups were calculated relative to the SD group (SD = 1).

### 2.11 Statistical analysis

Data are expressed as mean ± standard deviation (SD). For the comparison of experimental groups, two‐way ANOVA followed by Bonferroni's post hoc were used. For the analysis of PVAT+ and the respective PVAT− rings, an unpaired Student's *t*‐test was used (Instant Software, GraphPad Prism, USA). A value of *p* < 0.05 was considered statistically significant.

## 3 RESULTS

### 3.1 Exercise performance

After the moderate aerobic exercise program, we verified an improvement in the exercise capacity of TR and OB‐TR groups as assessed by the incremental tests. The animals in the TR group increased the performance measured by the total time (minutes) by 52%, distance (meters) by 102%, and maximal speed (m/min) by 43% when compared with the SD; the OB‐TR group increased these variables by 50%, 88%, and 39%, respectively, when compared with the OB‐SD group (Table 1). In contrast, the OB‐SD group exhibited a reduction in exercise capacity across all variables compared to the SD. When we compared only the trained groups, we also observed a decrease in exercise performance in the OB‐TR group compared with the TR group.

**TABLE 1:** Incremental exercise test performed at the end of the training program in female mice from the sedentary (SD), trained (TR), obese sedentary (OB‐SD), and obese trained (OB‐TR) groups.

	SD (14)	TR (14)	OB‐SD (14)	OB‐TR (14)
Time (minutes)	20.1 ± 3.6	30.6 ± 4.1^a^	12.3 ± 4.3^a^	18.5 ± 4.2^b^, ^c^
Distance (meters)	335.0 ± 97.7	678.5 ± 157.9^a^	162.2 ± 80.4^a^	306.0 ± 107.9^b^, ^c^
Max speed (m/min)	24.8 ± 3.8	35.6 ± 4.2^a^	16.9 ± 4.5^a^	23.6 ± 4.4^b^, ^c^

*Note*: Data are mean ± SD. The number of animals per group is indicated in parentheses. Two‐way ANOVA followed by Bonferroni post hoc test.

^a^ *p* <0.05 compared with SD.

^b^ *p* <0.05 compared with TR.

^c^ *p* <0.05 compared with OB‐SD.

### 3.2 Description of the experimental model

Initial body weight was similar across all experimental groups (Table 2). A significant difference in body weight in the OB‐SD and OB‐TR groups compared with the SD and TR groups, respectively, was observed after 2 weeks on a high‐fat diet (data not shown). After 8 weeks of the experimental protocol, the OB‐SD and OB‐TR groups showed an increase in body weight of approximately 60% compared to the SD and TR groups. By the end of the protocol (after 16 weeks), body weight in the OB‐SD and OB‐TR groups had increased by approximately 90% compared to their respective control groups fed standard chow. This reflects the greater weight gain in the OB‐SD and OB‐TR groups (Table 2).

**TABLE 2:** Body weight and weight gain in female mice from the sedentary (SD), trained (TR), obese sedentary (OB‐SD), and obese trained (OB‐TR) groups.

	SD (14)	TR (14)	OB‐SD (14)	OB‐TR (14)
I‐Body weight (g)	20.4 ± 1.3	20.3 ± 1.8	21.4 ± 2.6	20.7 ± 1.2
8 Weeks body weight (g)	23.9 ± 1.5	22.7 ± 1.4	38.3 ± 6.8^a^	36.5 ± 3.4^b^
F‐Body weight (g)	25.2 ± 2.1	23.6 ± 1.4	48.2 ± 4.9^a^	45.8 ± 6.2^b^
Weight gain (g)	4.8 ± 1.3	3.3 ± 1.1	26.8 ± 3.7^a^	25.1 ± 6.4^b^

*Note*: Data are mean ± SD. The number of animals per group is indicated in parentheses. Two‐way ANOVA followed by Bonferroni post hoc test.

^a^ *p* <0.05 compared with SD.

^b^ *p* <0.05 compared with TR.

Additionally, the pWAT and iBAT were greater in the OB‐SD and OB‐TR groups compared with the SD and TR groups. Moderate aerobic exercise training reduced only the iBAT in the OB‐TR group compared with the OB‐SD group. Animals fed a high‐fat diet had a higher daily caloric intake compared to control animals fed standard chow. In contrast, water consumption was lower in the OB‐SD and OB‐TR groups than in the SD and TR groups. No significant differences in systolic blood pressure (SBP) and heart rate (HR) were observed (Table 3).

**TABLE 3:** Food and water intake, final blood glucose, perigonadal white adipose tissue (pWAT) and interscapular brown adipose tissue (iBAT), systolic blood pressure (SBP), and heart rate (HR) in female mice from the sedentary (SD), trained (TR), obese sedentary (OB‐SD), and obese trained (OB‐TR) groups.

	SD (10)	TR (10)	OB‐SD (10)	OB‐TR (10)
Food intake (kcal/animal/day)	12.0 ± 0.6	11.9 ± 1.0	33.4 ± 7.7^a^	29.1 ± 5.9^b^
Water intake (mL/animal/day)	5.2 ± 0.5	5.1 ± 0.6	3.1 ± 0.5^a^	2.8 ± 0.6^b^
pWAT (mg/mm)	22.8 ± 4.4	21.9 ± 2.2	203.0 ± 8.1^a^	206.8 ± 17.1^b^
iBAT (mg/mm)	3.5 ± 0.4	2.5 ± 0.4	19.9 ± 1.8^a^	10.7 ± 1.2^b^ ^,^ ^c^
F‐Blood glucose (mg/dl)	87 ± 9.1	90 ± 13.8	139 ± 19.8^a^	124 ± 14.4^b^
F‐SBP (mmHg)	96 ± 6.9	98 ± 11.5	97 ± 9.8	97 ± 14.2
F‐HR (bpm)	681 ± 78.9	668 ± 38.0	646 ± 87.1	590 ± 62.2

*Note*: Data are mean ± SD. The number of animals per group is indicated in parentheses. pWAT and iBAT values were normalized by tibial length. Two‐way ANOVA followed by Bonferroni post hoc test.

^a^ *p* <0.05 compared with SD.

^b^ *p* <0.05 compared with TR.

^c^ *p* <0.05 compared with OB‐SD.

**FIGURE 2:**
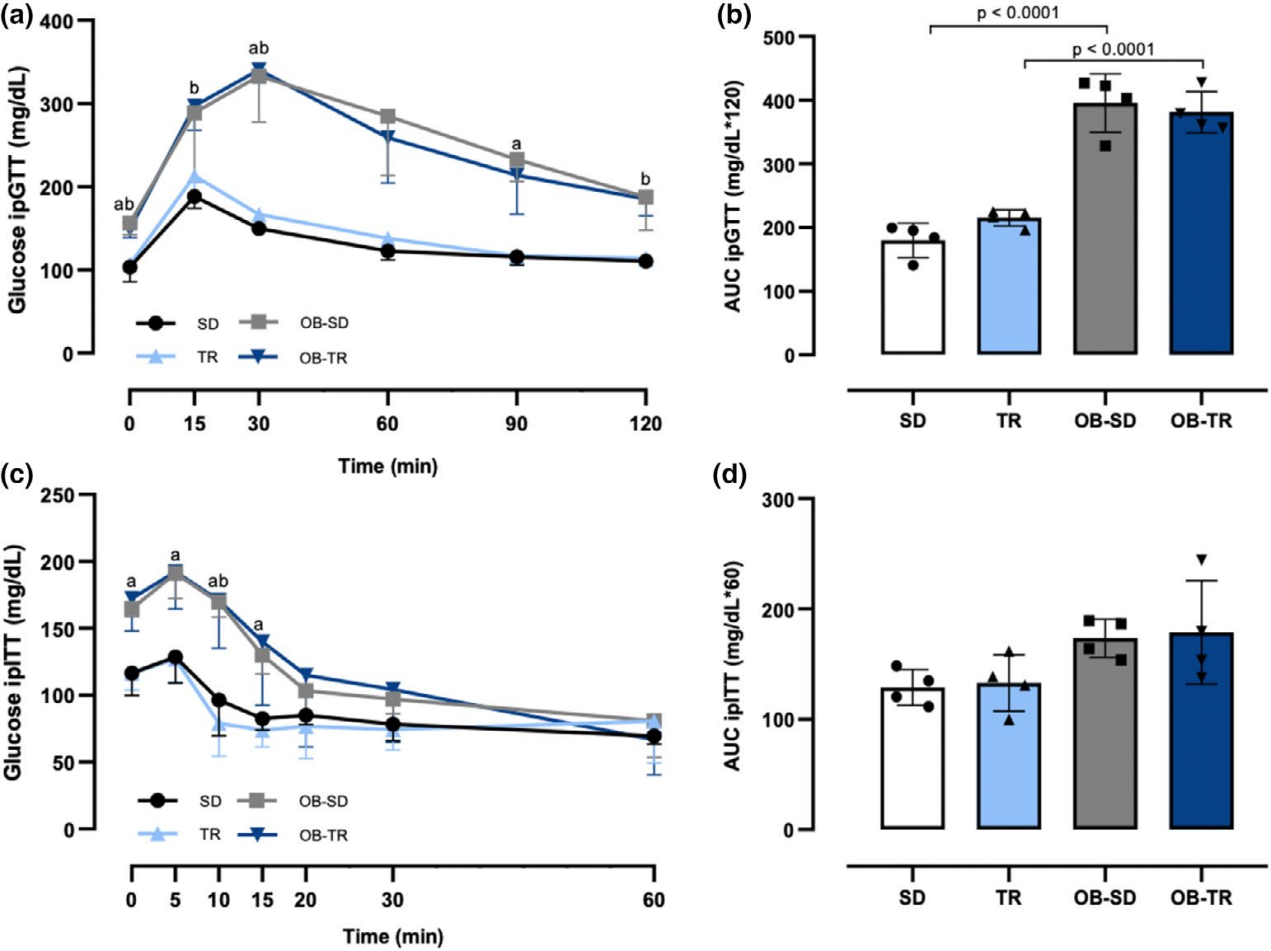
Glucose homeostasis was assessed by intraperitoneal glucose tolerance test (ipGTT) and insulin tolerance test (ipITT). Blood glucose before (0) and 15, 30, 60, 90, and 120 min after intraperitoneal glucose administration (ipGTT, panel a) and its area under curve (AUC, panel b). Blood glucose before (0) and 5, 10, 15, 10, 30, and 60 min after intraperitoneal insulin administration (ipITT, panel c) and its area under curve (AUC, panel d). Female mice from sedentary (SD), trained (TR), obese sedentary (OB‐SD), and obese trained (OB‐TR) groups. Data are presented as mean ± SD. Two‐way ANOVA followed by Bonferroni post hoc test. ^a^ *p* <0.05 compared with SD; ^b^ *p* <0.05 compared with TR. For the area under curve the *p* value is indicated in the figure.

Glucose tolerance and insulin sensitivity were impaired in the OB‐SD and OB‐TR groups, indicating altered glucose homeostasis in this experimental model. The ipGTT was performed after a 6‐h morning fast, and the OB‐SD and OB‐TR groups exhibited higher glucose levels throughout the test compared with the SD and TR groups (Figure 2a). Also, the area under the curve (AUC) values for ipGTT were increased in the OB‐SD and OB‐TR groups (Figure 2b). The ipITT was performed after a 2‐h morning fast, and the data showed that within the first 15 min after insulin administration, glucose levels in the OB‐SD and OB‐TR groups were higher compared with the SD and TR groups (Figure 2c), without changes in AUC values for ipITT (Figure 2d). In addition, 12‐h overnight fasting blood glucose levels increased by approximately 60% in the OB‐SD group compared to the SD group and by 37% in the OB‐TR group compared to the TR group (Table 3).

### 3.3 Systemic inflammation and oxidative stress

**FIGURE 3:**
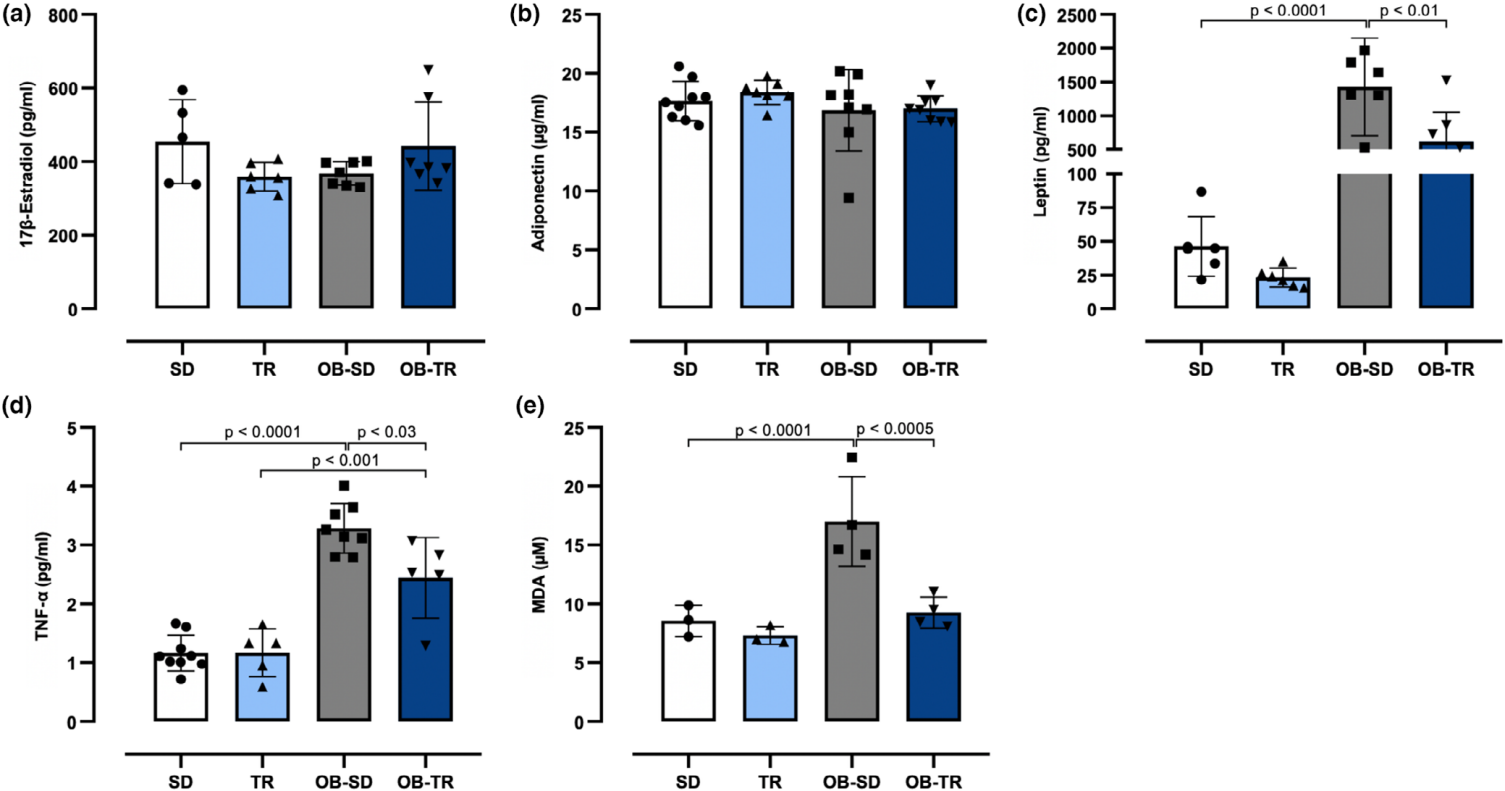
Serum levels of total 17β‐estradiol (panel a), adiponectin (panel b), leptin (panel c), TNF‐α (panel d), and malondialdehyde (MDA, panel e) in female mice from sedentary (SD), trained (TR), obese sedentary (OB‐SD), and obese trained (OB‐TR) groups. Data are presented as mean ± SD. Two‐way ANOVA followed by Bonferroni post hoc test. The *p* value is indicated in the figure.

There was no significant difference between the groups in serum 17β‐estradiol and adiponectin levels (Figure 3a,b). On the other hand, serum leptin concentration (Figure 3c) was increased in the OB‐SD and OB‐TR groups compared to the SD and TR groups. Exercise training effectively decreased leptin levels in the OB‐TR group compared to the OB‐SD group. TNF‐α level (Figure 3d) was elevated in the OB‐SD group compared to the SD group, and exercise training was able to partially reduce TNF‐α level in the OB‐TR group compared to the OB‐SD group. Lipid peroxidation is a well‐established mechanism of cellular injury and is used as an indicator of oxidative stress; thus, the malondialdehyde (MDA) production is an important biomarker in this context. In the OB‐SD group, serum MDA concentration (Figure 3e) was increased compared to the SD group, and exercise training mitigated this alteration in the OB‐TR group compared to the OB‐SD group.

### 3.4 Morphology and phenotype of tPVAT

**FIGURE 4:**
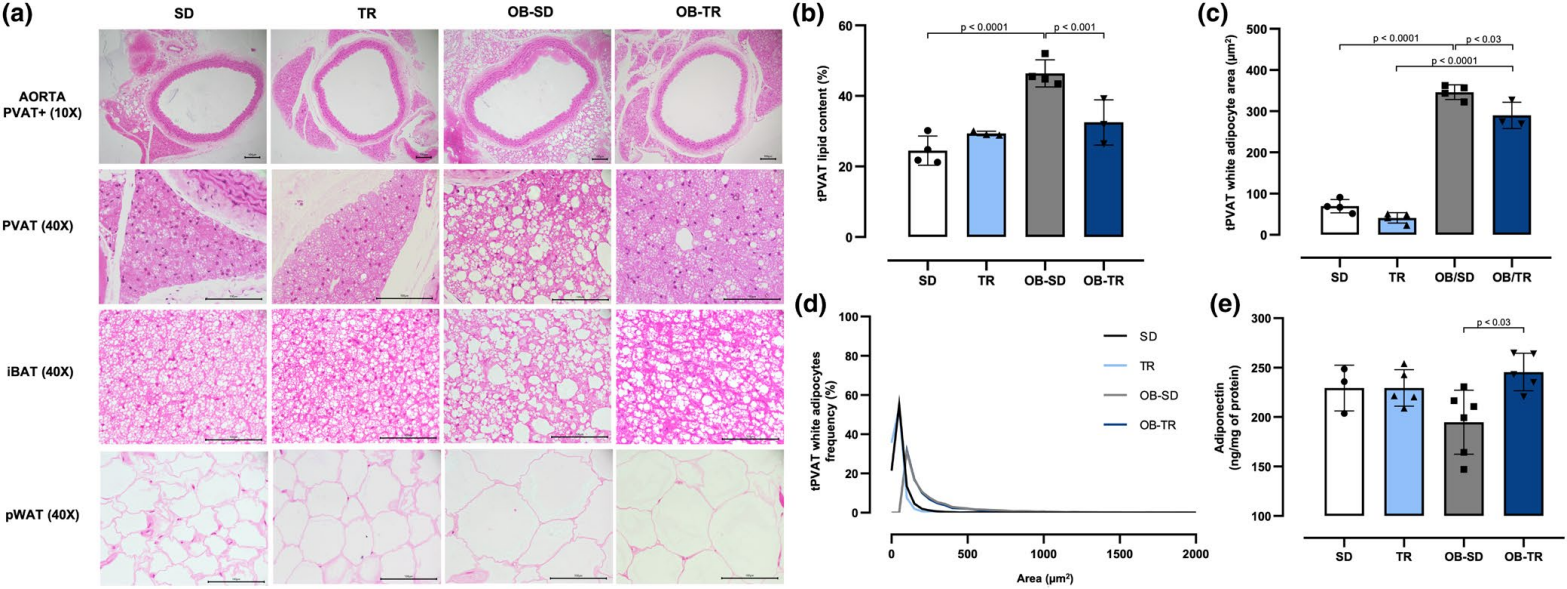
Qualitative and quantitative histological analyses were performed on female mice from the sedentary (SD), trained (TR), obese sedentary (OB‐SD), and obese trained (OB‐TR) groups. Representative hematoxylin–eosin photomicrographs of adipose tissue depots (panel a): Perivascular adipose tissue (tPVAT) from thoracic aorta, interscapular brown adipose tissue (iBAT), and perigonadal white adipose tissue (pWAT). Digital images were captured using 10× and 40× objectives and scale bar = 100 μm. Percentage (%) of total lipid content in tPVAT (panel b), area (μm^2^) of white adipocytes in tPVAT (panel c), frequency (%) distribution of white adipocyte areas in tPVAT (panel d) and tPVAT adiponectin levels (e). Data are presented as mean ± SD. Two‐way ANOVA followed by Bonferroni post hoc test. The *p* value is indicated in the figure.

High‐fat diet‐induced obesity promoted a profound alteration in the morphology and phenotype of the tPVAT (Figure 4a). The SD group exhibited typical tPVAT morphology (Figure 4a), characterized by a phenotype more similar to BAT, as can be compared with the interscapular adipose tissue (iBAT) of a well‐established BAT depot. In addition, a smaller proportion of white adipocytes was also observed, identified by a single lipid droplet and a nucleus displaced peripherally. The TR group showed a predominance of brown adipocytes, with the nucleus more centrally located and surrounded by smaller lipid droplets. Additionally, BAT is characterized by the abundant presence of mitochondria and blood vessels, then showing a darker coloration macroscopically. On the other hand, in the OB‐SD group, the predominance of WAT and coalescence of lipid droplets characterized the phenotype of adipocytes in tPVAT, with nuclei displaced to the periphery. The nuclear shape is predominantly flattened, although nuclei with altered shapes are observed depending on their phase of cellular differentiation. Exercise training was able to maintain the morphology and phenotype of the tPVAT. The OB‐TR group displayed brown adipocytes, and the adipocytes exhibited smaller lipid droplets than the OB‐SD group. Thus, aerobic exercise demonstrated the ability to mitigate morphology alterations in tPVAT resulting from high‐fat diet‐induced obesity.

Quantification analysis of adipocytes indicated that the tPVAT of the OB‐SD group presented an increased percentage of lipid content compared to the SD group (Figure 4b), while exercise training was effective in reducing the percentage of lipid content in the OB‐TR group compared to the OB‐SD group. The OB‐SD group also showed a larger area of white adipocytes (Figure 4c) compared to the SD group. In the OB‐TR group, the area of the white adipocytes was partly reduced compared with the OB‐SD group. The distribution of white adipocytes in tPVAT according to their area showed that the OB‐SD and OB‐TR groups had larger white adipocytes with a higher percentage of cells in the 100–250 μm^2^ range compared with the SD and TR groups, which predominantly contained cells in the 50–100 μm^2^ range (Figure 4d). The adiponectin content in tPVAT was significantly increased in the OB‐TR group compared with the OB‐SD group (Figure 4e).

### 3.5 Vascular contractile response

**FIGURE 5:**
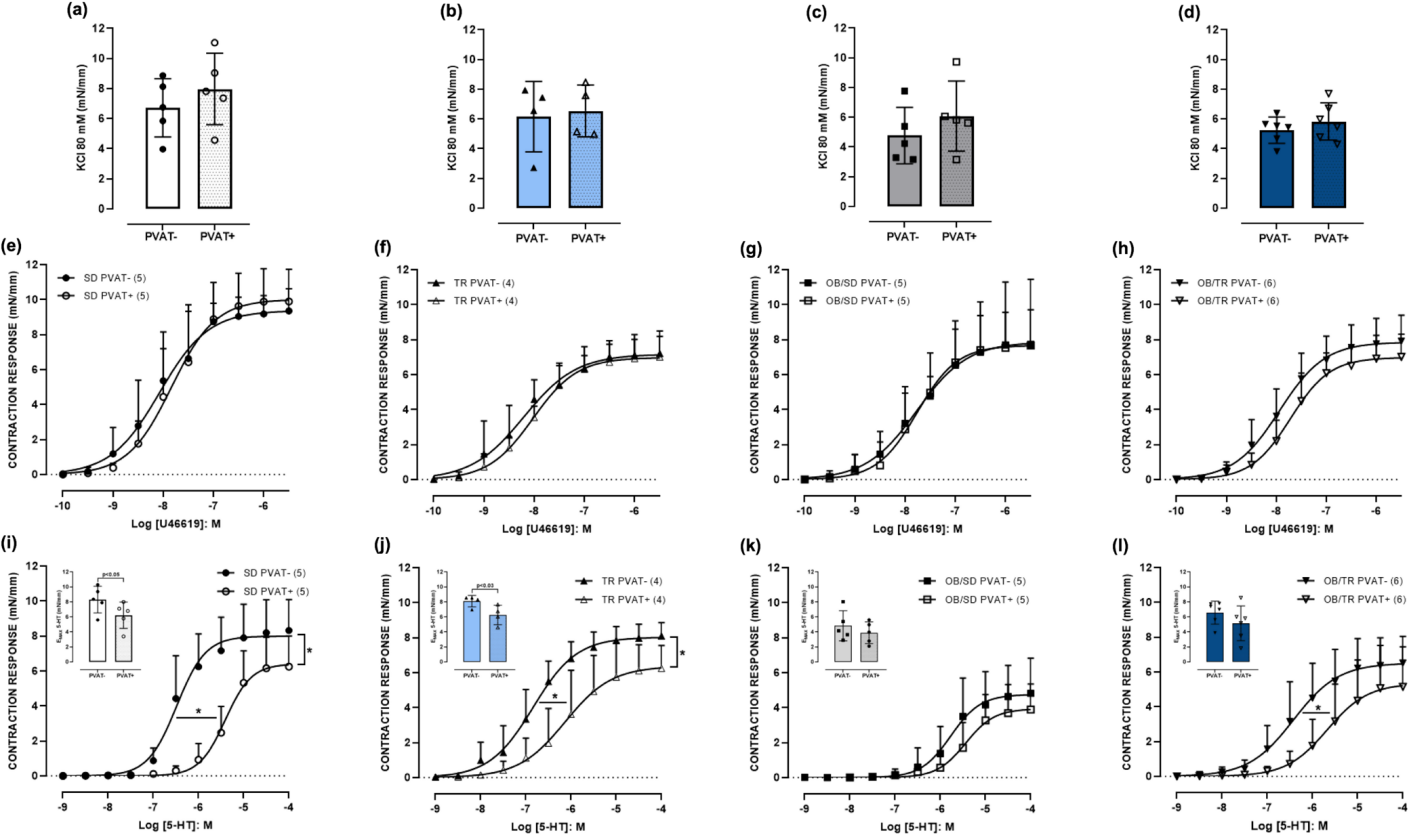
Maximal contractile response to KCl 80 mM (PVAT− and PVAT+) in thoracic aorta (panels a–d). Concentration–response curves to thromboxane A2 analogue (U46619; panels e–h) and serotonin (5‐HT; panels i–l) in thoracic aorta. Groups: sedentary (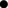
SD PVAT− and 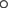
SD PVAT+), trained (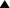
TR PVAT− and 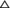
TR PVAT+), obese sedentary (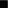
OB/SD PVAT− and 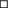
OB/SD PVAT+), and obese trained (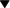
OB/TR PVAT− and 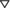
OB/TR PVAT+). Data are presented as mean ± SD. The number of animals per group is indicated in the figure. Student's *t*‐test: *p < 0.05 PVAT+ compared with the respective PVAT−. For the maximal response (E_MAX_) to serotonin (5‐HT) the *p* value is indicated in the figure.

The presence of PVAT did not change the contractile response to KCl 80 mM in the thoracic aorta with intact endothelium in all groups (Figure 5a–d). Neither the maximal response (E_MAX_) nor the potency (pEC_50_) was modified for thromboxane A2 analog (U46619) in PVAT+ rings compared with PVAT‐ rings in all groups (Figure 5e–h). On the other hand, the PVAT+ rings exhibited an anti‐contractile response to serotonin (5‐HT) with decreased pEC_50_ and E_MAX_ in SD (approximately 14.8‐fold, and 2.1 mN/mm) and TR (approximately 6.6‐fold, and 1.9 mN/mm) groups in comparison to the respective PVAT− rings (Figure 5i,j). In the OB‐SD group, the PVAT did not change the pEC_50_ and E_MAX_ to 5‐HT (Figure 5k). Aerobic exercise partly reestablishes the anti‐contractile response to 5‐HT in PVAT+ rings, with a reduction in pEC_50_ in the OB‐TR group (approximately 5.1‐fold) in comparison to the respective PVAT− rings (Figure 5l).

When we analyzed only the PVAT− rings, no alteration was observed to KCl 80 mM or U46619 in all groups (Table 4). On the other hand, the OB‐SD group exhibited hypocontractility to 5‐HT with decreased pEC_50_ and E_MAX_ (approximately 5.0‐fold, and 3.5 mN/mm) compared with PVAT− rings from the SD group, and this alteration was not observed in the OB/TR group (Table 4). When we analyzed only the PVAT+ rings, the vascular contractile response was similar in all groups for KCl 80 mM, U46619, and 5‐HT (Table 5).

**TABLE 4:** Maximal contractile response to KCl 80 mM, maximal response (E_MAX_) and potency (pEC_50_) obtained from concentration‐response curves to thromboxane A2 analog (U46619) and serotonin (5‐HT) in female mice thoracic aorta PVAT− from sedentary (SD), trained (TR), obese sedentary (OB/SD), and obese trained (OB/TR) groups.

PVAT−		SD (5)	TR (4)	OB/SD (5)	OB/TR (6)
KCl 80 mM		6.7 ± 1.9	6.2 ± 2.3	4.8 ± 1.9	5.2 ± 0.9
U46619	E_MAX_	9.3 ± 1.3	7.2 ± 1.0	7.7 ± 3.7	7.9 ± 1.4
	pEC_50_	8.11 ± 0.50	8.22 ± 0.70	7.96 ± 0.51	7.96 ± 0.36
5‐HT	E_MAX_	8.3 ± 1.7	8.1 ± 0.8	4.8 ± 2.0^a^	6.6 ± 1.5
	pEC_50_	6.47 ± 0.24	6.91 ± 0.36	5.77 ± 0.41^a^	6.38 ± 0.45

*Note*: Data are mean ± SD. The number of animals per group is indicated in parentheses. Potency is represented as –log of molar concentration to produce 50% of the maximal responses obtained from concentration‐response curves to U46619 and 5‐HT. Two‐way ANOVA followed by Bonferroni's post hoc.

^a^ *p* <0.05 compared with SD.

**TABLE 5:** Maximal contractile response to KCl 80 mM, maximal response (E_MAX_) and potency (pEC_50_) obtained from concentration‐response curves to thromboxane A2 analog (U46619) and serotonin (5‐HT) in female mice thoracic aorta PVAT+ from sedentary (SD), trained (TR), obese sedentary (OB/SD), and obese trained (OB/TR) groups.

PVAT+		SD (5)	TR (4)	OB/SD (5)	OB/TR (6)
KCl 80 mM		8.0 ± 2.4	6.5 ± 1.7	6.1 ± 2.3	5.8 ± 1.2
U46619	E_MAX_	9.9 ± 1.8	7.0 ± 1.5	7.7 ± 2.0	7.0 ± 1.3
	pEC_50_	7.80 ± 0.33	8.07 ± 0.28	7.77 ± 0.40	7.75 ± 0.25
5‐HT	E_MAX_	6.2 ± 1.8	6.2 ± 1.3	3.9 ± 1.4	5.1 ± 2.3
	pEC_50_	5.30 ± 1.22	6.09 ± 0.61	5.41 ± 0.26	5.67 ± 0.43

*Note*: Data are mean ± SD. The number of animals per group is indicated in parentheses. Potency is represented as –log of molar concentration to produce 50% of the maximal responses obtained from concentration‐response curves to U46619 and 5‐HT. Two‐way ANOVA followed by Bonferroni's post hoc.

### 3.6 Reactive oxygen species (ROS) and β‐galactosidase activity (β‐gal)

**FIGURE 6:**
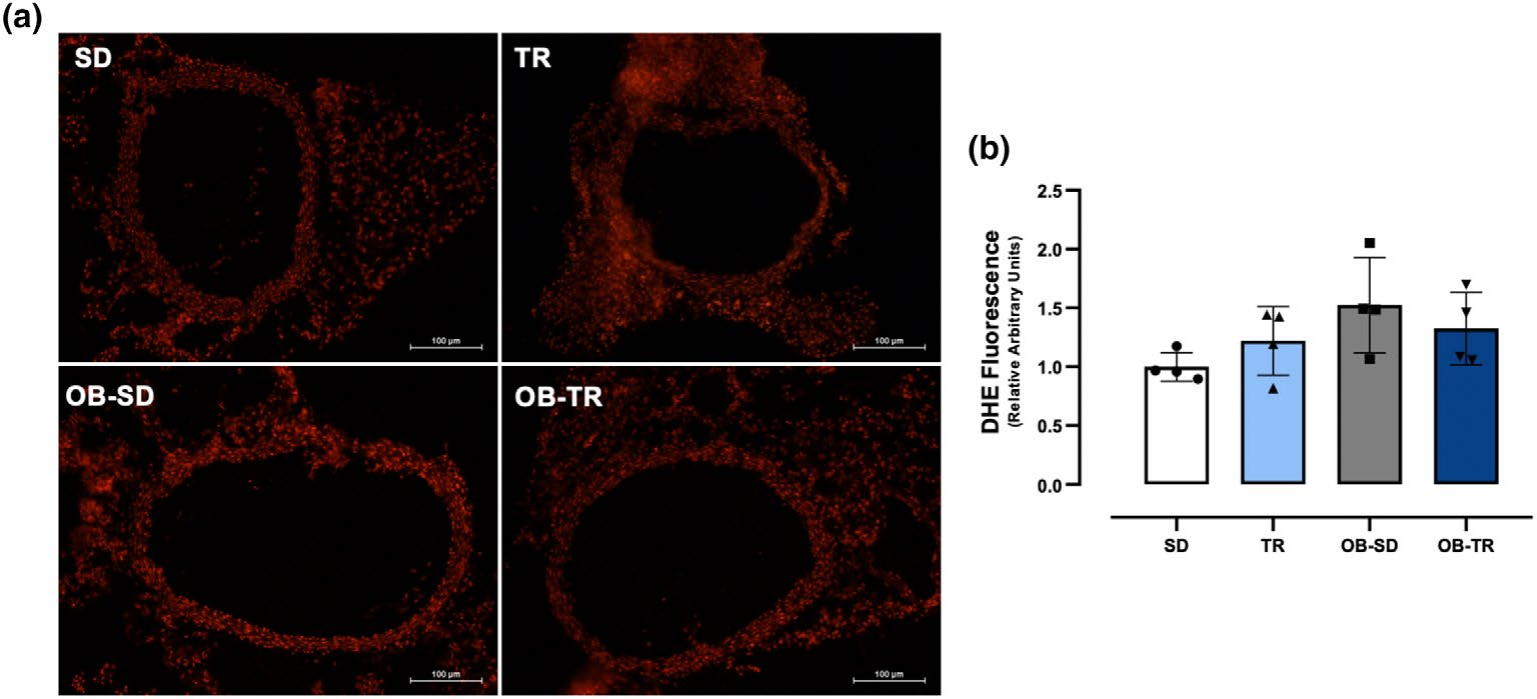
Reactive oxygen species (ROS) generation. Representative photomicrographs of the ethidium‐bromide‐positive nuclei in transverse sections of thoracic aorta and PVAT (panel a), from sedentary (SD), trained (TR), obese sedentary (OB‐SD), and obese trained (OB‐TR) groups. Quantitative analysis of the fluorescence (panel b). Data are presented as mean ± SD. Two‐way ANOVA followed by Bonferroni post hoc test.

For the determination of ROS on the thoracic aorta and tPVAT, slices were incubated with DHE (Figure 6a). The fluorescence quantification revealed no significant differences between the groups (Figure 6b).

**FIGURE 7:**
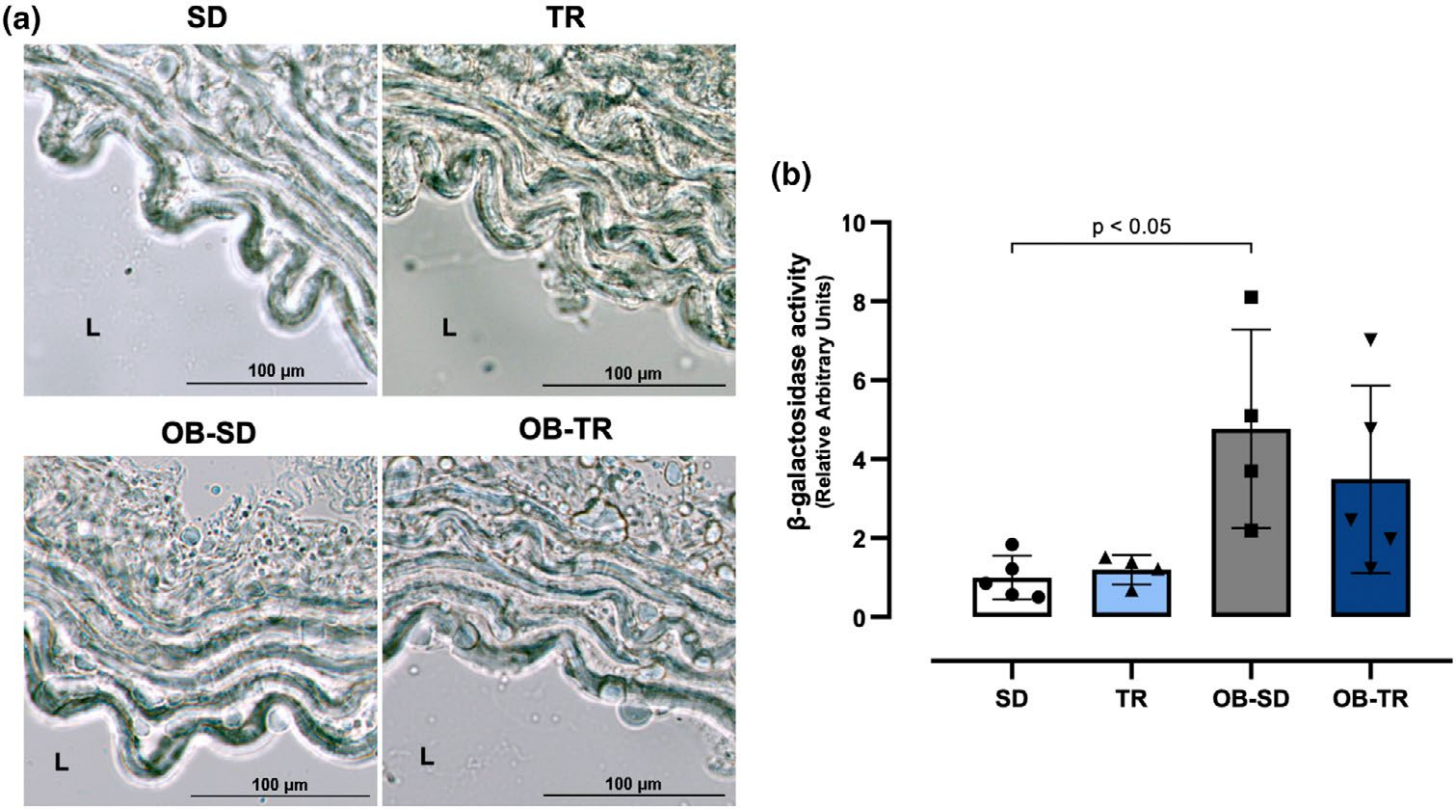
β‐galactosidase activity. Senescence‐associated β‐galactosidase activity in the thoracic aorta (panel a) from sedentary (SD), trained (TR), obese sedentary (OB‐SD), and obese trained (OB‐TR) female mice. Quantitative analysis of the blue staining intensity (panel b). L, Lumen. Data are presented as mean ± SD. Two‐way ANOVA followed by Bonferroni post hoc test. The *p* value is indicated in the figure.

The β‐galactosidase activity permits the identification of senescent cells in mammalian tissues (Figure 7a) Quantification analysis demonstrated that the OB‐SD group exhibits higher staining intensity for vascular senescent cells compared to the SD group. No significant differences were observed in the TR and OB‐TR groups, or between the OB‐TR and OB‐SD groups (Figure 7b).

## 4 DISCUSSION

In this study, we demonstrated that high‐fat diet‐induced obesity in female mice led to a significant phenotypic shift in tPVAT, transforming into a whiter phenotype, characterized by a unilocular morphology and enlarged lipid droplets. This change was associated with metabolic dysfunction, vascular cellular senescence, increased systemic inflammation and oxidative stress, and significant alterations in contractile response to 5‐HT. Aerobic exercise training effectively mitigated several obesity‐induced changes in tPVAT, preserving brown adipocytes with increased adiponectin and reducing circulatory inflammation and oxidative stress, and partially normalizing the contractile response. These findings highlight its importance as a non‐pharmacological intervention in obesity conditions in females.

Obesity, characterized by energy imbalance and excessive adipose tissue, is associated with cellular aging, chronic inflammation, and increased oxidative stress (Santos & Sinha, 2021). These alterations are important risk factors for developing secondary pathologies, such as CVD (Costa et al., 2018). In this study, high‐fat diet‐induced obesity was confirmed by the increase in body weight, perigonadal adipose tissue weight, and elevated blood glucose levels.

Aerobic exercise is recommended for maintaining or improving cardiovascular fitness (Garber et al., 2011). At the end of the moderate‐intensity training protocol, trained groups (TR and OB‐TR) exhibited improved physical performance compared to the sedentary animals (SD and OB‐SD). Similar outcomes were observed previously in our laboratory with male mice (Sousa et al., 2019). However, female mice achieved lower maximum speeds and had more difficulty completing the 8‐week program, particularly the obese animals.

Regular exercise helps alleviate the adverse consequences of obesity, including weight and body fat loss (Bigornia et al., 2010; Larson‐Meyer et al., 2010). However, consistent with other studies in male mice (Hespe et al., 2016; Petridou et al., 2019; Samaan et al., 2014), we did not observe changes in body weight and pWAT following the exercise training protocol. Furthermore, beyond the total quantity of adipose tissue, its integrity and functionality are more significant factors for cardiometabolic risk (Bastien et al., 2014; Koliaki et al., 2019; Sousa et al., 2019). Aerobic exercise also influences glucose homeostasis. A study in male mice showed that 6 weeks on a high‐fat diet‐induced insulin resistance, which was attenuated by 8 weeks of aerobic training, with insulin sensitivity assessed after a 6‐h fast (Muñoz et al., 2017). Another trial demonstrated in obese male mice that an intervention with 8 weeks of aerobic exercise significantly attenuated insulin resistance, following an 8‐h fast prior to the ITT (Muñoz et al., 2021). A recent study with obese male mice reported a significant improvement in GTT and ITT following 12 weeks of aerobic training and a 6‐h fast (Cao et al., 2024). In our study, the obese groups (OB‐SD and OB‐TR) also presented impaired glucose tolerance and insulin sensitivity; however, these parameters were not restored by 8 weeks of aerobic exercise in female mice. It is important to recognize that a limitation of our study is the 12‐h overnight fasting prior to the experiments and the possible changes in adipose tissue metabolism during fasting.

PVAT engages in the production of adipokines, and changes in its secretory profile, induced by obesity, can impair various physiological processes (Ketonen et al., 2010; Marchesi et al., 2009). Leptin is a lipostatic hormone synthesized primarily by white adipose tissue and, to a lesser extent, by brown adipose tissue (Gálvez‐Prieto et al., 2012). Importantly, its synthesis is proportional to the size of adipocytes (Stanek et al., 2021). Thus, as observed in our data, animals in the OB‐SD group have higher serum leptin concentrations; in fact, leptin may be one of the drivers of sympathetic nervous system hyperactivity in obesity (Xia et al., 2016). As demonstrated in this study, one of the effects of moderate‐intensity aerobic exercise was the reduction of leptin levels in the OB‐TR group. Other studies, including those from our research group, have shown in male rats and mice that moderate aerobic training can reduce the state of hyperleptinemia in obese animals (Sousa et al., 2021; Sponton et al., 2017; Wang et al., 2023). Similar to our results, a study in obese female mice showed reduced leptin levels when the animals were subjected to free wheel running (Yuksel Ozgor et al., 2020). The same study also found no differences in serum adiponectin levels between the groups, a finding observed in our study as well.

Obesity‐induced PVAT dysfunction causes inflammation, promoting immune cell infiltration (monocytes, macrophages, leukocytes, and dendritic cells) (Cheng et al., 2023). The M1 macrophage subclass secretes cytokines such as TNF‐α, IL‐6, and IL‐1, contributing to the inflammatory state (Stanek et al., 2021). Adipocyte hypertrophy leads to poor perfusion, local inflammation, and hypoxia, mainly mediated by HIF‐1α, which increases cytokine levels such as TNF‐α and IL‐6 (Agabiti‐Rosei et al., 2024). Our data showed that serum levels of TNF‐α increased in the OB‐SD group. A pro‐inflammatory profile was also observed in the tPVAT of male mice fed a high‐lipid diet (Ketonen et al., 2010; Omar et al., 2014; Xia et al., 2016), as well as in the serum of obese male rats (Sponton et al., 2017; Wang et al., 2023). Physical exercise has anti‐inflammatory effects and minimized immune cell infiltration in the epididymal adipose tissue of obese male mice (Kawanishi et al., 2010, 2013). Additionally, reducing adipocyte size decreases cytokine secretion such as TNF‐α and IL‐6 (Fritzen et al., 2015; Stanek et al., 2021). Accordingly, our results indicated that moderate‐intensity aerobic training reduced serum TNF‐α levels in the OB‐TR group. Before our study, no other study has evaluated TNF‐α serum levels in obese trained female mice; similar results were observed in male rats and mice using a moderate‐intensity aerobic training protocol (Sousa et al., 2019; Sponton et al., 2017; Wang et al., 2023).

The inflammatory environment established in obesity exacerbates the production of ROS. In this regard, the TNF‐α inhibitor infliximab effectively reduced mitochondrial hydrogen peroxide (H_2_O_2_) levels in the tPVAT of obese male mice. Furthermore, the lack of TNF‐α receptors improved tPVAT modulation of vascular contraction in these animals (da Costa et al., 2017). Our data demonstrated an increased serum MDA concentration in the OB‐SD group, although DHE fluorescence revealed no significant differences in the thoracic aorta and tPVAT. Other studies with male animal models also observed oxidative stress conditions (Costa et al., 2018; Gil‐Ortega et al., 2014; Ketonen et al., 2010; Xia et al., 2016). Moderate‐intensity aerobic exercise improves the antioxidant defenses and, therefore, effectively reduces ROS (Man et al., 2023; Roque et al., 2013). In our study, the serum oxidative stress biomarker was reduced in the OB‐TR group. In line with our data, a study with diabetic male mice showed that aerobic training increased the expression of antioxidant enzymes (Lee et al., 2011). Additionally, in hypertensive male rats, moderate treadmill running normalized superoxide production (Roque et al., 2013). Few studies address this topic in females. One demonstrated that volitional exercise prevented oxidative stress in the myocardial interstitial space (Bostick et al., 2017), while another revealed that high‐intensity interval training reduced oxidative stress in skeletal muscle (Pimenta et al., 2015). Importantly, we demonstrate that aerobic exercise training reduced serum MDA levels, a biomarker of oxidative stress, in obese female mice.

The PVAT phenotype is dependent on its vascular bed (Fitzgibbons et al., 2011; Police et al., 2009), and it is widely accepted that tPVAT is morphologically and functionally similar to BAT. As observed in our study, other studies in male mice and rats have described tPVAT as displaying multilocular brown adipocytes and abundant mitochondria (Fitzgibbons et al., 2011; Padilla et al., 2013; Victorio et al., 2020). The phenotypic alteration in tPVAT adipocytes, through specific signaling pathways, is evident in the tissue's whitening and browning. As presented in our data, obesity increases the lipid content of tPVAT and a white phenotype characterized by a unilocular morphology and enlarged lipid droplets in the OB‐SD group. A previous study showed similar results in lipid content in male rats (Liao et al., 2021). Other studies have also shown, in obese male mice, the predominance of white adipocytes in tPVAT (Gálvez‐Prieto et al., 2012; Sacks & Symonds, 2013). Aerobic exercise promotes beneficial changes in tPVAT morphology in male obese rats (Wang et al., 2023). In obese female mice, our study provides the first evidence of the predominance of smaller adipocytes with a brown‐like phenotype and reduced lipid content in the OB‐TR group. Similar results were observed in other studies with male rats, in which moderate‐intensity aerobic physical training reversed the increase and coalescence of lipid droplets in tPVAT and mesenteric PVAT (mPVAT) of obese animals (Araujo et al., 2018; Liao et al., 2021; Wang et al., 2023).

As previously stated, PVAT secretes adiponectin, an important anti‐inflammatory adipokine involved in various physiological processes and crucial for maintaining vascular homeostasis (Sowka & Dobrzyn, 2021). In obesity, adiponectin signaling is also dysregulated. Adiponectin levels are decreased in tPVAT samples from different obesity models in male animals, such as ob/ob mice (Agabiti‐Rosei et al., 2024) and diet‐induced obese mice (Chatterjee et al., 2009; Wang et al., 2023). Our study is the first to evaluate adiponectin levels in the tPVAT of obese female mice. We demonstrate that, in obesity, aerobic exercise training increases adiponectin levels in tPVAT.

In obesity, the anti‐contractile role of PVAT is altered; however, few studies conducted in obese female rodents have demonstrated that PVAT may only partially retain its function. A study in female Dahl SS rats fed a high‐fat diet showed that only the potency to PHE was reduced in PVAT+ aortic rings (Watts et al., 2021). Another study demonstrated that the anti‐contractile effect of mPVAT in response to PHE is reduced by a high‐fat diet in female mice (Victorio et al., 2021). In our study, we demonstrated that the thoracic aorta PVAT− rings from OB‐SD exhibited hypocontractility to 5‐HT, and PVAT+ rings lost the anti‐contractile response, highlighting a significant alteration in vascular regulation in response to this agonist in female mice. No significant differences were observed in the concentration‐response curves to U46619.

The agonists 5‐HT and U46619 induce vasoconstriction by activating 5‐HT2A and thromboxane prostanoid receptors (TP), respectively, on VSMCs (Jiang et al., 2021; Machida et al., 2013; Ni et al., 2004, 2008). These receptors stimulate phospholipase C (PLC) via Gq/G11‐proteins, leading to the production of inositol triphosphate (IP3) and diacylglycerol (DAG), which increase intracellular calcium (Ca^2+^) and activate protein kinase C (PKC) (Hoyer et al., 1994; Yan et al., 2019). Additionally, the Gα(q/11) subunit can activate Dbl family RhoGEFs, enhancing RhoA signaling (García‐Redondo et al., 2015; Rojas et al., 2007). The PLC/IP3 and RhoGEF/RhoA pathways promote vasoconstriction by activating myosin light chain kinase and inhibiting myosin light chain phosphatase (Matsuo et al., 2011). Conversely, 5‐HT can also induce vasodilation via a NO‐dependent mechanism involving 5‐HT1B receptors on ECs. Activation of these receptors stimulates eNOS, leading to NO production from L‐arginine. NO then diffuses into VSMCs, where it activates soluble guanylate cyclase and increases cyclic GMP (cGMP) levels. cGMP activates protein kinase G (PKG), which lowers intracellular Ca^2+^ and promotes smooth muscle relaxation (Gamoh et al., 2013; Hoyer et al., 1994). Therefore, 5‐HT may function as either a vasoconstrictor or vasodilator depending on the vascular bed, reflecting differences in the distribution of 5‐HT receptor subtypes. Further studies should be performed to investigate the underlying mechanisms in 5‐HT vascular response in obesity and the role of PVAT.

In obesity, the burden of senescent cells in adipose tissue, pancreas, liver, and brain can increase, regardless of chronological age (Narasimhan et al., 2022; Palmer et al., 2022). These cells begin to secrete inflammatory factors, proteases, and growth factors, characterizing a SASP (Palmer et al., 2022). In our study, we found that β‐galactosidase activity in the thoracic aorta was increased in the OB‐SD group. Other studies have investigated cellular senescence, with one revealing that chronic hyperinsulinemia, commonly associated with obesity, induced the premature onset of the SASP in both subcutaneous and omental adipose tissue, observed in vitro or humans (Li et al., 2021). Another study, in humans with severe obesity, demonstrated that β‐galactosidase activity is correlated with serum leptin levels, insulin resistance, and increased body fat mass (Rouault et al., 2021). Additionally, exercise was observed to reduce the β‐galactosidase activity in male mice fed a high‐fat diet and subjected to voluntary exercise on a running wheel (Schafer et al., 2016). In our training protocol, no changes were observed in β‐galactosidase activity in the OB‐TR group. Further investigation is warranted, particularly in female‐specific protocols.

For a long time, both basic and clinical research overlooked sex specificities in the obtained results. There was a tendency to treat males and females as equivalent, leading to the predominance of males in experimental designs. While recognizing the importance of research in both sexes, a limitation of the present study is the exclusion of the male cohort; our decision was based on the previous studies performed in our laboratory involving a similar protocol in male mice (Sousa et al., 2019, 2021) and the absence of studies involving female mice.

Our study using obese female mice revealed a significant phenotypic and functional alteration in tPVAT, which was linked to increased vascular senescence, metabolic dysfunction, systemic inflammation, and lipid peroxidation. Moderate‐intensity aerobic exercise training attenuated these morphological changes, reducing circulating inflammation and oxidative stress, and partially restoring the anti‐contractile response to 5‐HT, even without changes in body weight and glucose homeostasis. Recognizing the biological differences between males and females in relation to obesity and its associated complications is essential, as it offers valuable insights into the mechanisms underlying obesity and improves our understanding of the condition in females as well.

## AUTHOR CONTRIBUTIONS

TMDR was involved in conceptualization, methodology, software, formal analysis, investigation, data curation, writing—original draft, and visualization. GAS was involved in methodology and investigation. LHR was involved in investigation. CBRV was involved in validation and investigation. SRC was involved in conceptualization, formal analysis, investigation, and supervision. MAD was involved in conceptualization, formal analysis, investigation, resources, data curation, writing—original draft, writing—review and editing, supervision, project administration, and funding acquisition.

## FUNDING INFORMATION

This work was supported by Fundação de Amparo à Pesquisa do Estado de São Paulo (FAPESP) grants 2022/09111‐9 to MAD and 2022/10354‐3 to TMDR; Fundo de Apoio ao Ensino, Pesquisa e Extensão (FAEPEX) grant 2062/23 to MAD; Coordenação de Aperfeiçoamento de Pessoal de Nível Superior (CAPES) grant 88887.712583/2022‐00 to TMDR; Conselho Nacional de Desenvolvimento Científico e Tecnológico (CNPq) grant 157401/2022‐7 to TMDR.

## CONFLICT OF INTEREST STATEMENT

The authors declare no conflicts of interest.

## ETHICS STATEMENT

Animal study was approved by the Ethics Committee for Animal Use (CEUA 5849‐1) at the Universidade Estadual de Campinas (UNICAMP), as stated by the Brazilian Society of Laboratory Animal Science.

## Data Availability

Data supporting this study are available upon request to the corresponding author.
